# Plant microbe based remediation approaches in dye removal: A review

**DOI:** 10.1080/21655979.2022.2049100

**Published:** 2022-03-16

**Authors:** Ekambaram Gayathiri, Palanisamy Prakash, Kuppusamy Selvam, Mukesh Kumar Awasthi, Ravindran Gobinath, Rama Rao Karri, Manikkavalli Gurunathan Ragunathan, Jayaprakash Jayanthi, Vimalraj Mani, Mohammad Ali Poudineh, Soon Woong Chang, Balasubramani Ravindran

**Affiliations:** a Department of Plant Biology and Plant Biotechnology, Guru Nanak College (Autonomous), Chennai - 600 042, India; b Department of Botany, Periyar University, Periyar Palkalai Nagar, Salem 636011, India; c College of Natural Resources and Environment, Northwest A&F University, Taicheng Road 3#, Yangling, Shaanxi 712100, PR China; d Department of Civil Engineering, SR University, Warangal 456, India; e Faculty of Engineering, University Teknologi, Brunei, Asia; f Department of Advanced Zoology and Biotechnology, Guru Nanak College, Chennai, India; g Department of Agricultural Biotechnology, National Institute of Agricultural Sciences, Rural Development Administration, Jeonju 54874, Korea; h Department of Food health, Islamic Azad University, Moazen Blvd, Karaj, Iran; i Department of Environmental Energy and Engineering, Kyonggi University, Youngtong-Gu, Suwon 16227, Republic of Korea

**Keywords:** Phytoremediation, dyes remediation, microbes, degradation textile, plant

## Abstract

Increased industrialization demand using synthetic dyes in the newspaper, cosmetics, textiles, food, and leather industries. As a consequence, harmful chemicals from dye industries are released into water reservoirs with numerous structural components of synthetic dyes, which are hazardous to the ecosystem, plants and humans. The discharge of synthetic dye into various aquatic environments has a detrimental effect on the balance and integrity of ecological systems. Moreover, numerous inorganic dyes exhibit tolerance to degradation and repair by natural and conventional processes. So, the present condition requires the development of efficient and effective waste management systems that do not exacerbate environmental stress or endanger other living forms. Numerous biological systems, including microbes and plants, have been studied for their ability to metabolize dyestuffs. To minimize environmental impact, bioremediation uses endophytic bacteria, which are plant beneficial bacteria that dwell within plants and may improve plant development in both normal and stressful environments. Moreover, Phytoremediation is suitable for treating dye contaminants produced from a wide range of sources. This review article proves a comprehensive evaluation of the most frequently utilized plant and microbes as dye removal technologies from dye-containing industrial effluents. Furthermore, this study examines current existing technologies and proposes a more efficient, cost-effective method for dye removal and decolorization on a big scale. This study also aims to focus on advanced degradation techniques combined with biological approaches, well regarded as extremely effective treatments for recalcitrant wastewater, with the greatest industrial potential.

## Graphical abstract

**Figure:**
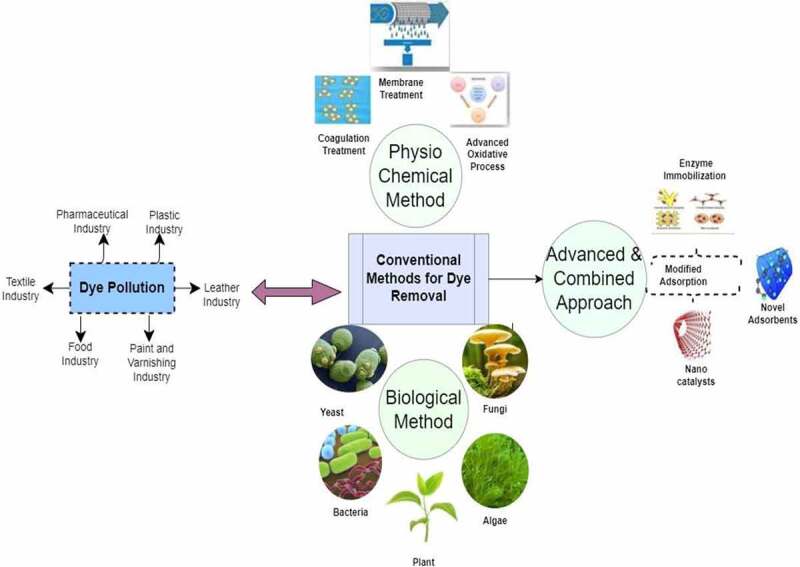

## 1. Introduction

Textile industry contributes significantly to global environmental degradation by the emission of unfavorable textile effluent. Textile wastewater comprises colors and a variety of pollutants in varying concentrations [1-4]. With increased pollution and environmental concern, scientists concentrated on these issues, since major water contamination issues not only cause health issues but also social issues [5]. As a result, environmental regulations often require textile mills to remediate effluents before discharging them into receiving waterways. The rapidly developing industrial sector particularly the textile industry (85%), is a source of harmful synthetic chemicals discharged mostly in the form of toxic dyes [6,7]. Globally, almost 80% of wastewater is not properly treated [8]. It is imperative to note that approximately 10–15% of synthetic colorants have oncogenic or mutagenic properties that pose detrimental effects on all living form [1,9–11]. Water sources that are vital for drinking, agriculture and for further purposes like domestic and industrial needs are now been contaminated by textile colors discharged into wastewater [12]. Every large-scale treatment effectiveness may be determined by feeding the system either with actual textile wastes or with synthetic wastewater with properties similar to those found in normal textiles manufacturing discharge.

Discharging textile toxic chemicals into river systems modifies the critical properties of the aquatic environment by affecting the BOD, COD, TSS, TOC, TDS, color and pH [13–16]. This ultimately leads to the formation of stink and a deterioration of the reservoir’s water quality [17]. Textile dyes’ resistance to breakdown in soil and water is a result of their complex chemical structure [18]. Textile effluents include reactive dyes including triazine that may cause cancer, birth abnormalities, and hormone disruption. Electrochemical degradation of azo reactive dye was shown to be beneficial in minimizing the formation of carcinogenic compounds during biodegradation [19,20]. Textile wastewater contains unfixed colours, inorganic and organic compounds, and trace metals that are toxic to the environment and may result in bleeding, vomiting, dermatitis illnesses, tumors, and genomic instability [21]. Hazardous chemicals’ endurance in aqueous and soil habitats may result in their buildup in plankton, fish, and plants. Similar to textile industry effluents, municipal sewage is also a major contaminant that has been released in water bodies [22]. Due to the limitations of both inorganic and organic materials, scientists are now focusing on the natural materials like bacteria, algae, fungi and actinomycetes for development of more active and safe materials for dye degradation [23]. Phytoremediation is a more efficient and cost-effective method of treatment than traditional methods. It makes use of the root systems of plants to absorb nutrients from wastewater. Plant species used for phytoremediation have the capacity to accumulate a narrow or broad spectrum of contaminants [24,25]. The objective of this review is to assess potential of several approaches for dye bioremediation. The methods of removal and the roles of microorganisms in the removal process are evaluated critically. In addition, a comprehensive analysis of important literature data on effluent properties, as well as substances, such as chemicals used to manufacture simulated sewage water, including dye, and treatments used to treat the generated effluents, were explored. Finally, the current state of knowledge about bioremediation of textile dyes is presented, along with recommendations for strategies enhancement and scientific advancement.

## 2. Review of literature methodology

**Figure 1.**
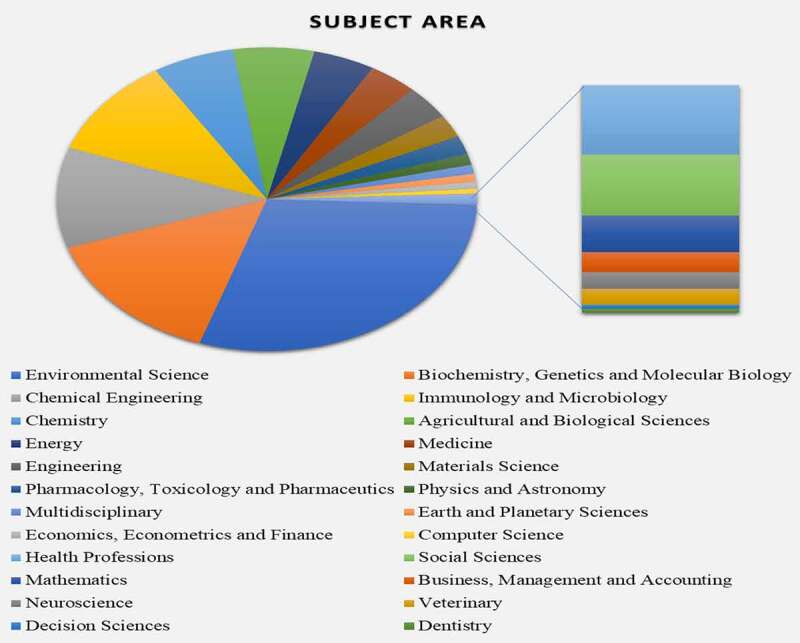
Summary of research papers published between 2000 to 2021 on dye bioremediation.

The relevant literature using the keywords “bioremediation of dyes” was search (as on May 2021) in Scopus, Google Scholar, and Science Direct to understand the significance of this research in present era. Figure 1 show the different subject areas, where dye bioremediation is used. The results were narrowed for the last 2 decades by specifying a time range ranging from 2000 to 2021. Figure 1 summarizes the number of papers describing dye bioremediation from 2000 to 2021. It may be seen that the number of papers on bioremediation of textile effluents has increased in recent years.

## 3. Dyes

Dyes are a class of chemicals that are often used in textiles. They are chemically synthesized or derived from plants and animals (Table 1). They are unique in that, unlike paint, they do not accumulate on the surface of the fiber but are absorbed into the molecule<apos;>s holes. This is conceivable for two reasons: -

- To begin, the dye molecules are smaller than the pores in the fiber.
- The dye molecules resemble narrow strips of paper in terms of length and width but have a comparatively thin thickness.

When the fiber, yarn, or cloth is placed into the dye bath, their planar form facilitates them in slipping into the polymer system. The main aspect is the dye’s attraction for the fiber is due to their attraction forces. The dye that has diffused or penetrated into the fiber is kept fixed in place because of the dye’s adhesion to the fiber [26,27]. According to a recent survey, about 100,000 dyestuffs are available commercially and about one million tons of dyes are manufactured yearly, with around 10% of dyes being dumped within the realm of natural assets as waste [28]. So, the dye removal from the waste water of the cosmetic, plastic, textile and paper industries is a current area of research in environmental protection. The majority of synthetic colors are non-biodegradable and poisonous [29,30]. Their potential pollution of water sources in the vicinity of dye-based industry raises environmental concerns [31,32].

There are around twenty-five different kinds of dyes depending on their chromophore’s chemical structure [33,34]. There are over a thousand dyes designated as textile dyes that are used for dyeing in wide range of clothing and accessories [35,36]. There are also several intermediates in the dyeing process that acts as a precursor to dyes. They can be produced using basic materials such as naphthalene and benzene through a wide range of chemical processes [37].

**Table 1.** Natural dyes obtained from Plant and Animal

Source	Natural Dyes	Derived from	Colorant	Chemical Structure	Application
Plant	Alkannin	*Alkanna tinctoria*	Purple	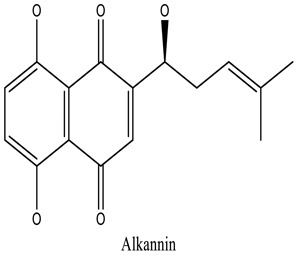	Cosmetics, soaps and pigments.
Plant	Brazilin	*Caesalpinia echinata* *Caesalpinia sappan*	Bright red	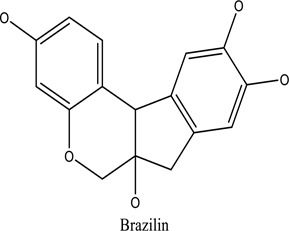	Cotton, wool
Plant	Rhamnetin	*Rhamnus petiolaris Bois*	Yellow to green organic colorant	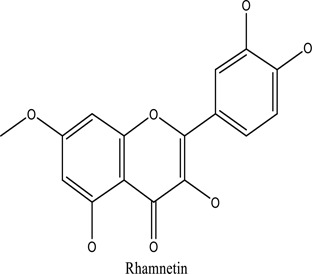	Textile industry
Plant	Quercetin	*R.cartharticus*	Bright yellow	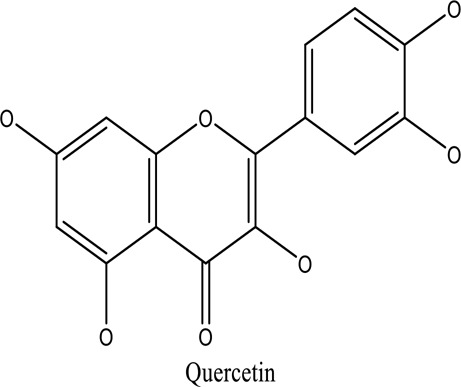	Textile industry
Plant	Chamomile	*Anthemis tinctoria*	Dark yellow	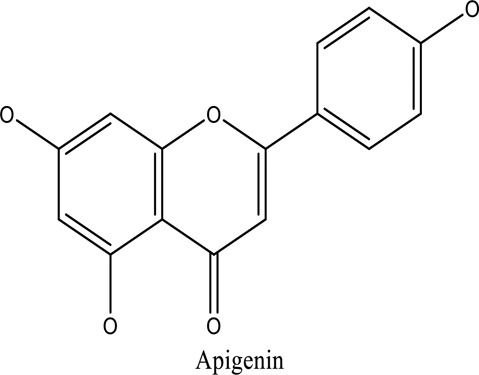	Textile industry
Plant	Chestnut	*Castanea sativa*	Brown	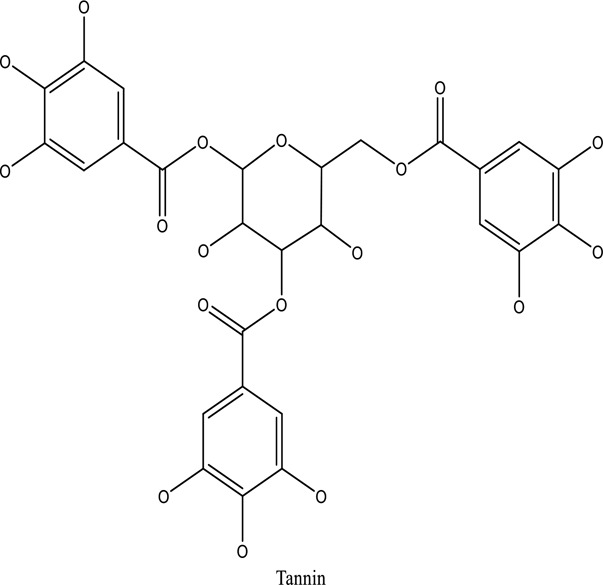	Textile industry
Plant	Cutch	*Acacia catechu*	Reddish brown	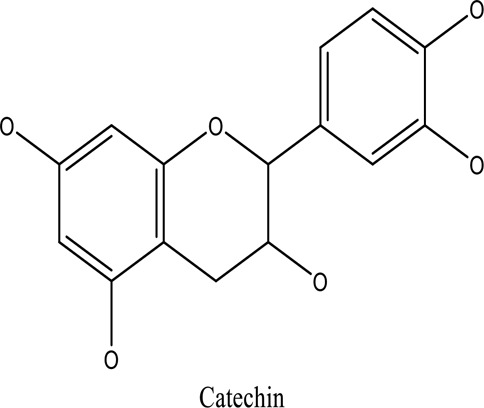	All Dyeing Industries
Animal	Cochineal	*Dactylopius coccus*	Red	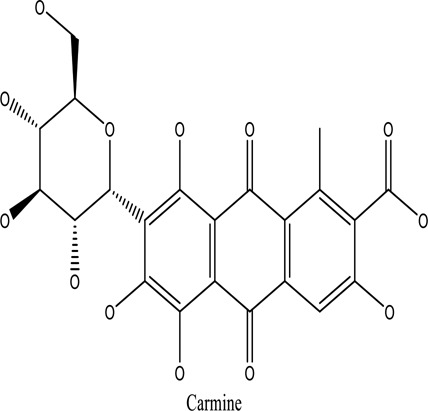	Food and in lipstick
Animal	Lac	*Kerria lacca*	Bright red	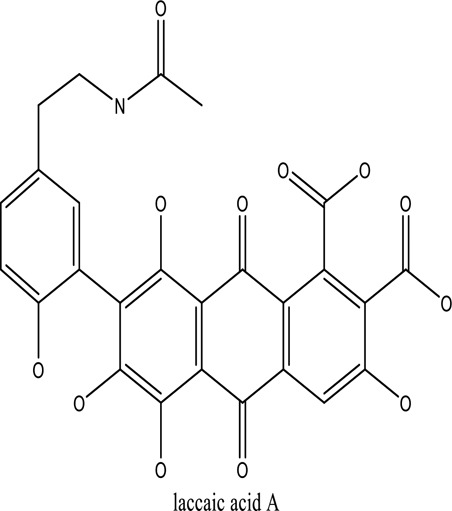	Textile industries
Animal	Tyrian	*Chicoreus palmarosae*	Reddish-purple	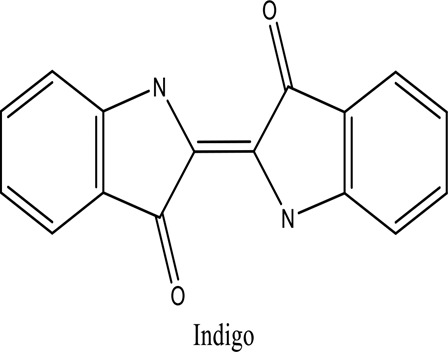	Textile industries
Animal	Sepia	*Sepia apama*	Reddish-brown	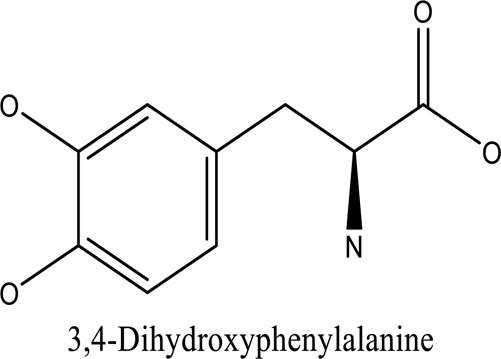	Pigment in Writing, Art and Cosmetics

### 3.1. dye classification

There were just a few natural dyes available prior to the introduction of synthetic dyes. As a result of the growth in the yearly world output of dyes, categorization of dyes has become necessary. They are expected to be in the range of many tens of millions of tonnes [38]. Dyes may be classified into a variety of classes depending upon its source, colour, structure, and manner of absorption (Figure 3: Textile dyes classification according to their structure).

### 3.2. Impact of dyes

The existence of colors in sewage at extremely low quantities is very apparent and undesired [39]. Over one lakh synthetic dye are in market with average production of 7 × 10^5^ tonnes colorants are synthesized yearly [40,41]. If exposed to light, water, or to any stress many complex dyes will not fade [42,43] Because of the complexity of their structural configuration and origin, dyes are difficult to decolorize. There are several structural variants, including acid, alkaline, dispersion, aldehyde, diazo, and anthroquinone-based dyes. When municipality drainage systems process textile dye wastewater aerobically, minimal decolonization occurs [44]. ETAD is a worldwide organization launched in 1974 with member companies located around the world. Its mission is to protect the environment. Members must follow the ETAD Code of Ethics, which is based on the principles of ethical treatment. They must also follow all national and international chemical rules [45]. ETAD has tested approximately four thousand dyes, which had a higher LD50 value of 2 × 10^3^ mg/kg. Basic and diazo direct dyes are considered as most hazardous dyes that considered as major mutagens to the all living organism.

### 3.3. Effect of dyes on health

**Figure 2.**
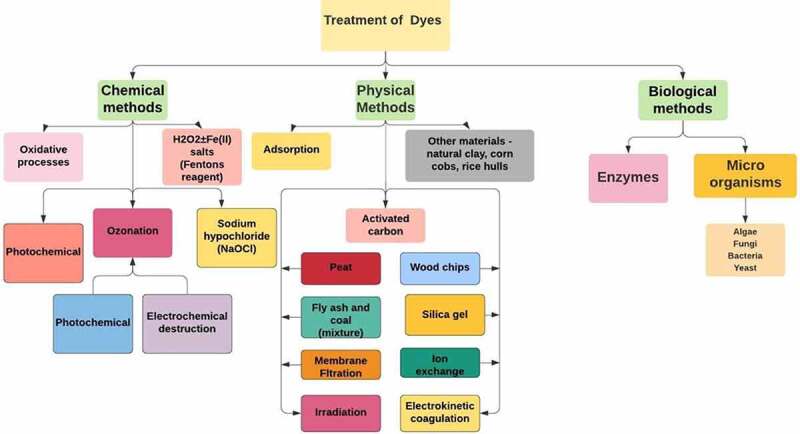
Methods in Dye treatment in Industries.

Sewage contains a range of toxic azo dyes and other organic contaminants. The hazardous contaminants are often discharged into the surroundings through a number of industries, including medicines, dyes, chemical synthesis, plastics, and petrochemicals. Numerous studies have focused on these toxic compounds due to their detrimental effects because of their toxicity which directly or indirectly affects all organisms [46–49]. Nitrophenols irritate the eyes and cause skin necrosis. Additionally, nitrophenols are toxic to all the major organs, mainly the kidney. Exposure to 4-nitrophenol, in particular, produces a variety of health issues in humans, including vomiting, sleepiness, migraines, and tachypnoea, through inhalation or ingestion, because of its cytotoxic, embryotoxic, oncogenic, and mutagenesis properties [50,51]. The majority of artificial azo dyes have a complex structure containing mono-di- azo dyes that exhibit severe allergic reactions when released in the ecosystem. It may ultimately cause mutation in different body parts [52–54] Diazo dyes, such as Congo red and Bismarck brown R, contain two azo groups and are very oncogenic and genotoxic. Additional consequences of azo dyes in water bodies include lower penetration of light into the water and decreased oxygen levels, both of which have an influence on the development of aquatic creatures and biota owing to lower photosynthetic activity. As a consequence, several governments have outlawed the use of azo dyes, while many nations continue to use those [55,56]. (Figure 2)

**Figure 3.**
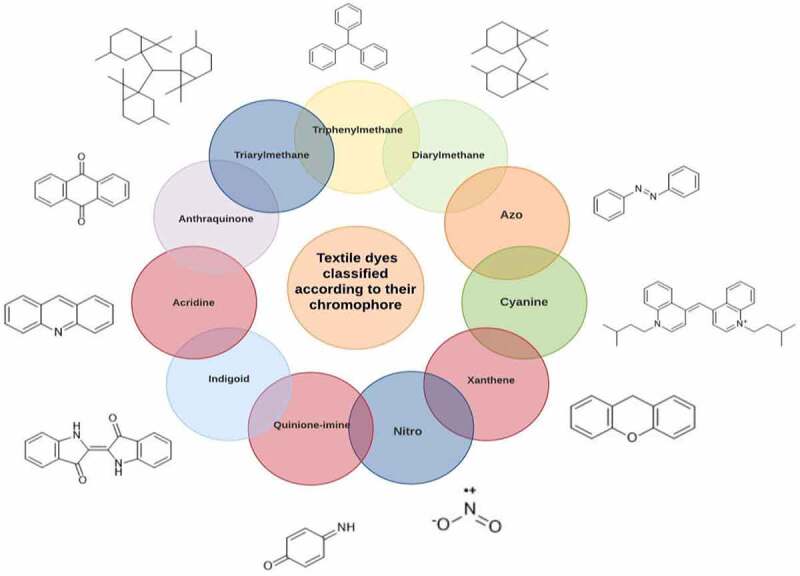
Textile dyes classification according to their structure °.

## 4. Biomaterials as adsorbent

Eliminating dye waste by traditional biodegradation procedures is unsuccessful because none of the textile chemicals are biodegradable **[**57,58**]**. Physicochemical processes such as membrane separation, filtering, chemical oxidation, and coagulation are cost-effective **[**59,60**]** (Figure 2). While adsorption techniques incorporating active carbons are shown efficacy in removing colors from industrial wastewaters, they are also rather costly **[**61,62**]**. There has been a surge in research interest in recent years on the sorption capabilities of bio-waste materials of plants and animals in regulating contaminants. These biomaterials, in conjunction with other biological processes, are demonstrating promising as a better solution to currently used techniques of remediation and recovery of ions of high value derived from wastewater wastes waterways **[**61,62**]**. Laccases may be used to decolorize textile effluents in bioremediation **[**62**]**. Such biological techniques have sparked an explosion and the research scientists started exploring biomaterials types that can be used as bioremediator in the various industrial sectors. The efficacy of many biomaterials shows promising results to mention a few: fly ash **[**63**]** modified calcined diatomite **[**64**]** unburned carbon **[**65**]** sand **[**66,67**]** Chitosan beads (Cdstari 2008), sugarcane bagasse **[**68,69**]** plasma-treated synthesized polyester fibers in removing synthetic dyes **[**70**]** and Mango stone **[**71**]** peanut husk **[**72**]**, date stones **[**73**]**, citrus limetta peel **[**74**]**, and oil palm **[**75**]** have all been documented, because to their availability & regenerative character, as well as their active functional groups such as hydroxyl and carboxyl groups **[**76**]**. Over the last decades, many studies have been elicited in the sorption potentials of solid waste of flora and fauna origin, either in their original condition or chemically altered forms, with the purpose of regulating harmful contaminating ions in waste waters. These techniques are shown themselves to be viable alternatives to conventional and traditional ways of pollution avoidance, spurring ongoing and expanded study in this sector

## 5. Biological methods

Biological approaches, namely the breakdown of dyes by biological processes like phytoremediation, are a low-cost, high-efficiency approach for removing dye from textiles discharge [77]. Biological material like algae, bacteria, fungus, and yeasts that can degrade and remove a variety of synthetic colors [78]. Phytoremediation-based techniques have indeed been effectively employed to degrade textile industry wastewater. Especially compared to other approaches, biological treatment (i.e., bioremediation) is cost-effective, environmentally beneficial, and creates less sludge [79]. It results in the oxidation of reactive polymers to a less hazardous inorganic product (i.e., chromophoric group) which ultimately aids in eliminating toxic compounds [80]. Recently adsorption of synthetic dyes was being analyzed by using a synergistic plant-microbe combination that maintains a sequential anaerobic-aerobic phase [81]. Synthetic dye like azo dye degradation takes place by a two-step procedure: first, the dyes are broken down to generate aromatic amines, and then the aromatic amines are further hydrolyzed to generate tiny non-toxic compounds in aerobic condition [82,83]. The strategies are been designed to reap the benefits of bacteria’s ability to survive both in aerobic and anaerobic environments in order to completely degrade the azo linkages produced inside the dyes. Microorganisms are effective at lowering COD and turbidity but ineffective at eliminating color [84,85]. So, in the coming decades, the usage of biological approaches for color removal may include the first phase as anaerobic processes and the second stage as aerobic processes [86].

### 5.1. Phytoremediation

**Figure 4.**
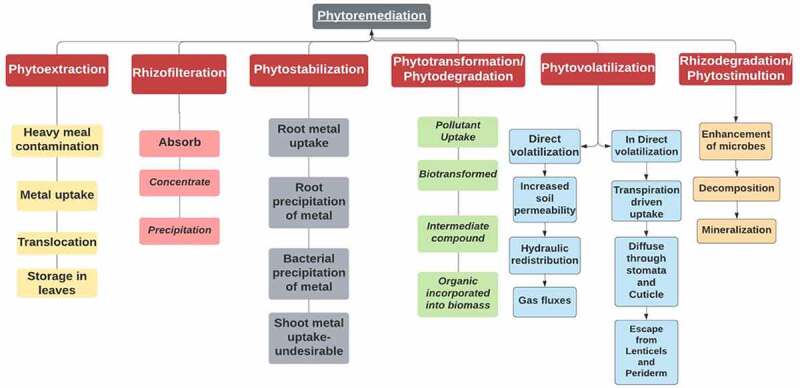
Classification of Bioremediation.

In the past two decades, phytoremediation has gained popularity as an environmentally benign, cost-effective, and complimentary technique to other readily available remediation methods [87–89]. Based on the properties and hydrophilicity of the pollutant, plants using one of two techniques to deal with them. They either collect pollutants in their cell organelles or breakdown to form intermediate metabolites or CO_2_ and water via enzymatic systems [90,91]. Antioxidants from plants that are not enzymatic and have two critical properties namely phenols and flavonoids. The composition of both substances has been attributed to their capability in eliminating generated Reactive oxygen species under stressful circumstances due to their redox characteristics that enable them to operate as singlet oxygen quenchers [92]. The need for plant species and microbes to neutralize and detoxify textile dyes as well as at the contaminated site definitely sounds to be a promising solution [93,94]. Phytoremediation is a renewable energy-based remedial technique that utilizes flora to decontaminate polluted places. Plants retain and stabilize toxins via their intrinsic enzymatic and absorption systems (Figure 4).

### 5.2. Green remediation of dyes

Green chemistry seems to be an appropriate prospective method of treatment of pollutants; it is accepted for its renewable energy sources, low cost, give the most accurate and can be used directly in polluted sites due to its long-term degradation potential. Many indigenous plants have been offered for dye removal, including *Typhonium flagelliforme, Phragmites australis, Rheum rabarbarum* (rhubarb), *Blumea malcolmii*, and *Rheum hydrolapatum* [95–99]. Similarly, *Glandularia pulchella, Tagetes patula,Petunia grandiflora, Aster amellus, Zinnia angustifolia* and *Portulaca grandiflora* are prepared flora in dye degradation [100–107]. In warmer climates, the use of *L. minor* Linn. favored the elimination of the Basic Red 46. Diverse species, including *Scirpsu grossus, Tecoma stans var. angustata*, aquatic plant *Spirodela polyrrhiza* and *Eichhornia crassipes* (water hyacinth), have also been considered for their dye degradation capability. For their function in dye biodegradation, a consortium of *P. grandiflora* and *G. grandiflora* plants has been created. Additionally, a combining method using plant-associated microorganisms in combination with *M. sativa* L. and *S. cannabina* Pers. has been suggested.

Some native species, such as *B.malcolmii, T. flagelliforme, R. hydrolapathum,R. rabarbarum*, and *P. australis* were used to treat nylon effluent [108–112]. Aquatic plants are capable of discoloration and detoxify dye-containing effluent. They were employed in dye degradation experiments at the laboratory scale and *in situ* [113,114]. Aquatic macrophytes like *Ammannia baccifera, Typha domingenesis Paspalum scrobiculatum Fimbristylis dichotoma, Ipomoea aquatica, Alternenthera philoxeroides, Typha angustifolia, Phragmites australis* and *Salvinia molesta* have recently been used as a decolorizer in a variety of manmade pollutants [115]. Most developing nations have used HRTS practices to achieve zero discharge from industrial dyes via the growth and maintenance species [116]. Plants such as *Accasia mangium, Dalbergia sisoo, Azadirachta indica*, and Eucalyptus sp. have the ability to degrade a huge quantity of pollutants. The contaminants adsorbed through trees are later evaporated into the atmosphere through stomatal pores [117]. Even though the usage of blooming and decorative plants seems appealing, their dye removal efficiency in the site is still to be validated. *Panicum virgatum* has reported that they have the ability to break down popular herbicides like atrazine [118]. Vetiver grass, mustard and tomato, and have all been shown to absorb EtBr from polluted locations [119]. *Salix viminalis* and *B. juncea* have shown the ability to phytoremediate polycyclic aromatic hydrocarbon-contaminated areas [120]. The use of *S. portulacastrum, T. vulgaris, R. officinalis, B. juncea* and T. *angustifolia* was investigated for *in situ* waste water treatment at artificial wetland and known to be potential species for dye removal. However, field implementation of phytoremediation continues to encounter a number of challenges, including the pollutants’ bioavailability, absorption, phytotoxicity, and evapotranspiration [121,122]. (Table 2: List of plant with structure and mechanism of the dyes).

**Table 2.** Plant in remediation of dyes

Plant	Dyes	Structure and formula weight	Mechanism			Reference
*T. erecta L*. *T. ammi L* *H. rosa-sinensis L* *C. indicum L* *B.fedtschenkoi* *C. roseus L*	Triarylmethane dye	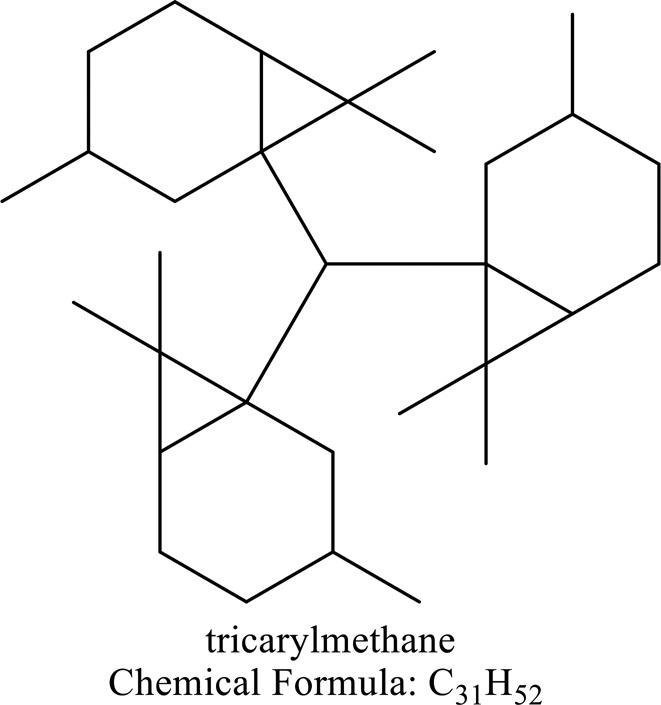	80% Efficiency in removing MB and CR dyes from inorganic dye			[Navjeet Kaur 2021.]
*C. sativa L*	Benzo α pyrene and chrysene	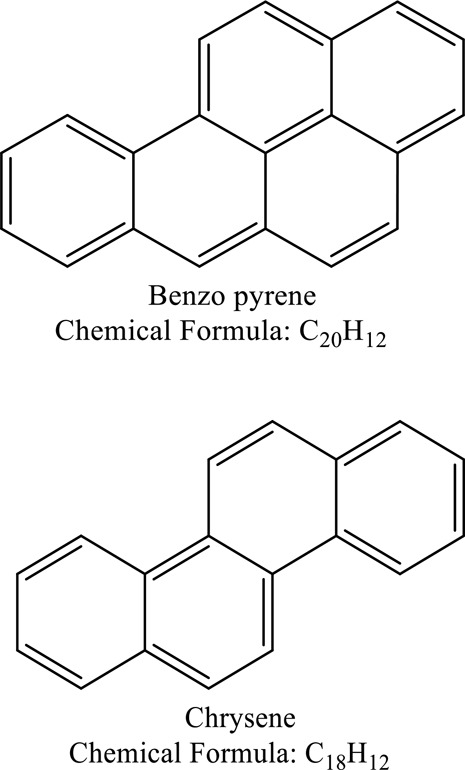		These species remove hazardous hydrocarbons from effluents dumped region producing a high microbial activity	[Campbell S, et al. (2006), Sanjeev Kumar 2017]	
*Glandularia pulchella*	3-ethylbenzothiazoline-6-sulphonic acid, n-propanol	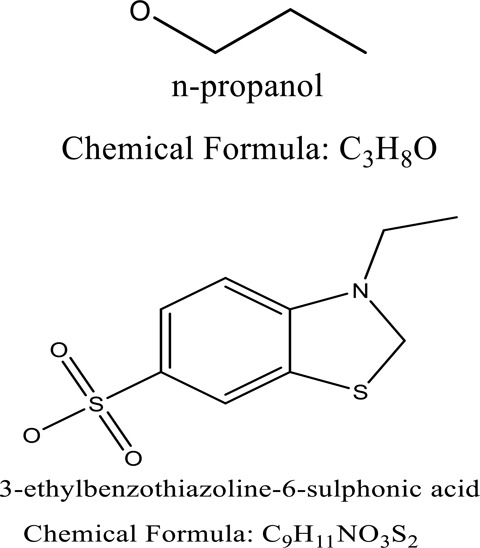		Improved degradation of tyrosinase, and 2,6-DCIP reductase	[Kabra, A.N 2011]	
*T. flagelliforme*	Reactive Red 2 Methyl Orange	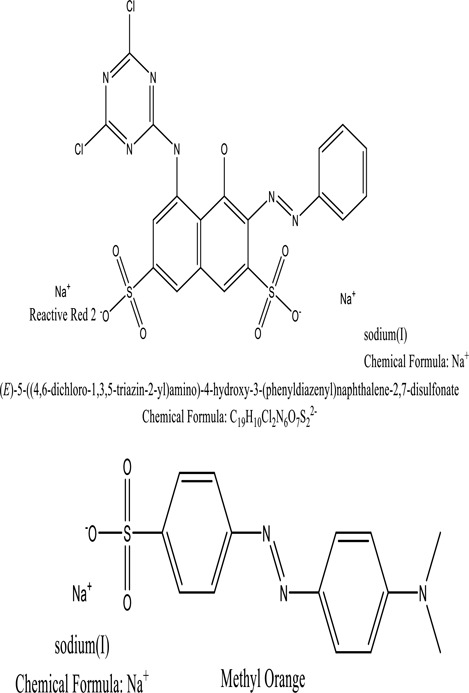		Potential species for the removal of Phenol, indophenol reductase	[Kagalkar, A.N 2010]	
*Aster amellus*	Remazol Red RB-133	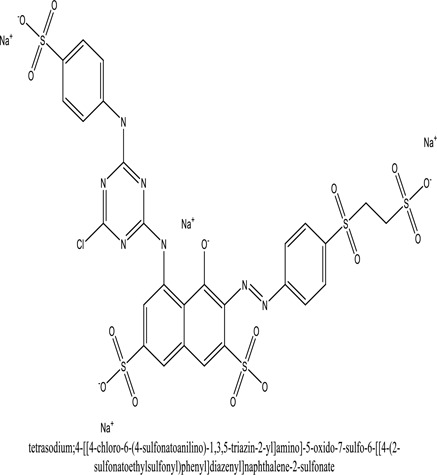		Improvement in the activity of oxidase, myeloperoxidase, veratryl methanol monoxide & methylene reductase.	[Khandare, R.V. 2011a]	
*Petunia grandiflora and Gaillardia grandiflora*	Brilliant Blue G	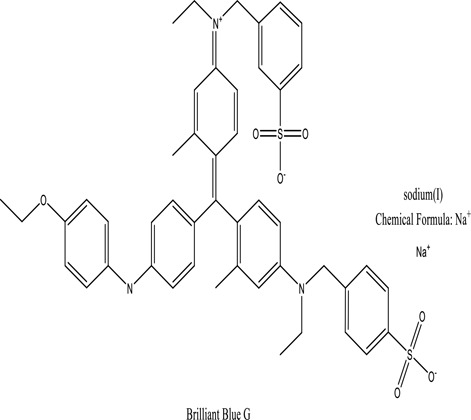		Laccase, Veratryl alcohol oxidase tyrosinase, and lignin activity were determined	[Watharkar, A. et al. 2014]	
*Nopalea cochenillifera*	Reactive Red 141	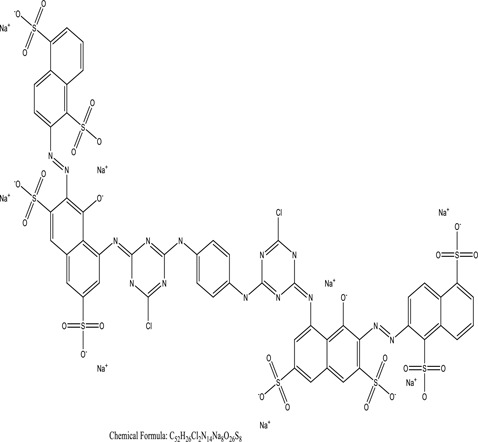		Removal of 2,6- DCPIP reductase	[Adki, V.S 2012]	
*Cucurbita pepo*	Direct Yellow DY106	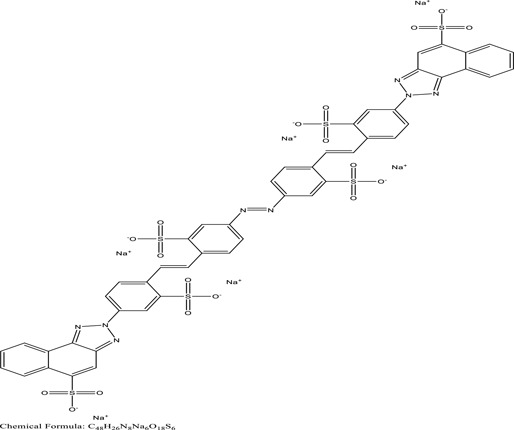		Extracted peroxidase	[Boucherit, N et al. 2013]	
*Portulaca grandiflora*	Reactive Blue 172	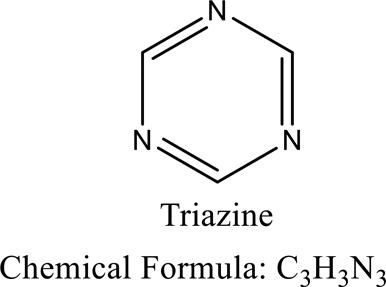		Increased activity of lignin oxidase, tyrosinase and DCPIP reductase	Khandare, R.V et al. 2011a	
*Eucalyptus sheathiana*	Basic Violet 10	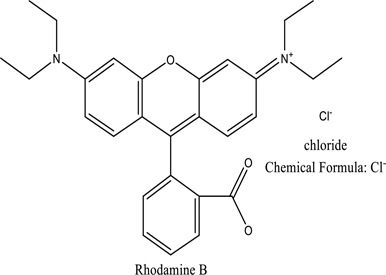		Achieved maximum adsorption level	Kooh, M.R.R 2016	
*T. ivorensis*	Direct Red 28	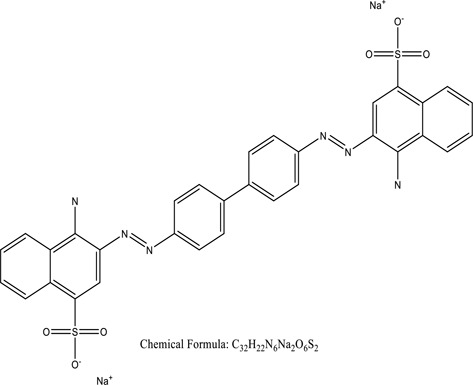		Maximum adsorption of MB and CR	Babalola, J. O., et al. 2016	
*Thymus vulgaris L, Rosmarinus officinalis L*	Allura red AC	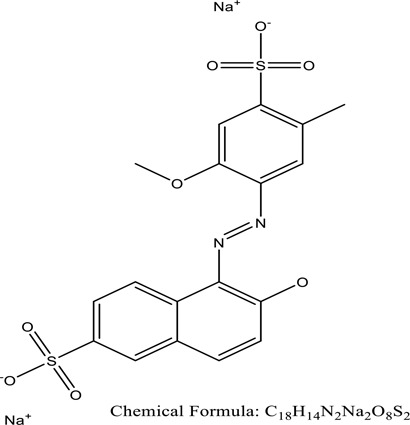		peroxidase activity	Zheng, Z., et al. (2000)	
*Prescaria barbata*	Reactive black 5	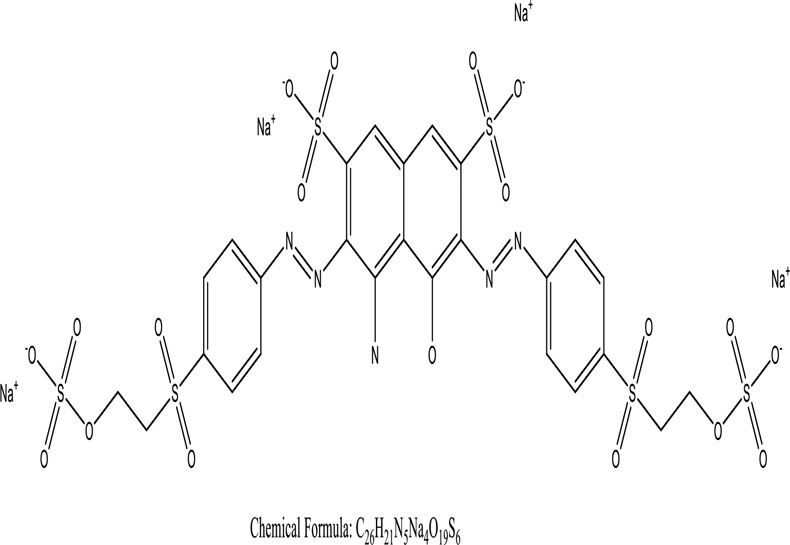		50% dye removed in the adsorption	Saba, B., et al. 2015	
*Blumea malcolmii Hook*	Malachite Green	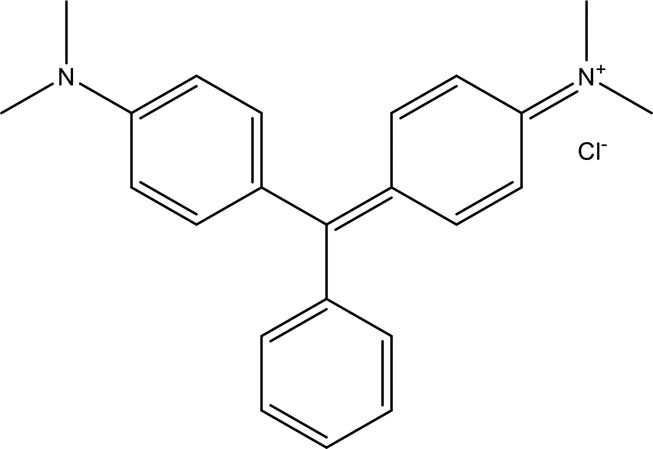		Decrease in the BOD, COD and ADMI values	Anuradha N. Kagalkar et al. 2011	

### 5.3. Remediation of dye using plant-microbe synergism

One among recent techniques in phytoremediation is that using the plant species and microbes (synergism) that are indigenous in marshes and upland areas over hundreds of years may prove to be more effective at cleanup. Plants’ root systems disseminate microbes throughout the ground surface and aid in their penetration of such impenetrable subsoil. Metabolic by-products from the roots promote the survivability and activity of microbes, resulting in an even more effective breakdown of contaminants [123]. Microbes either increase the bioavailability of contaminants to plants or minimize their cytotoxicity. Therefore, a synergism approach may increase the efficacy of phytoremediation. Several research on the synergistic removal of pollutants by flora and microorganisms have been documented. The elimination of PAHs and TPHs was enhanced in *F. arundinacea* by inoculating with rhizobacterial cultures [124,125]. *Thlaspi caerulescens* rhizospheric bacteria were inoculated in the roots, which resulted in a threefold rise in zinc concentration and a fourfold increase in zinc accumulation in shoots [126]. Studies show *Bacillus subtilis* SJ-101 promotes nickel building up in *Brassica juncea* [127].*B. subtilis* is an suitable strain that has alkaline pectinase properties, which is unique parameter for pretreatment of waste water from both paper and fabric industries [128]. In aquatic circumstances, *O. intermedium* BN-3 stimulated lead (Pb) absorption in the woody *E. camaldulensis* [129]. The synergy between *P. nigra* and *P.putida* has been shown to be highly efficacious in degrading diesel oil [130]. The consortial activity of *Z. angustifolia* and *E. aestuarii* ZaK resulted in a much more effective breakdown of the dye Remazol Black B [131]. (Table 3: List of plant-microbe synergism with structure and mechanism of the dyes).

**Table 3.** Plant Microbe synergism in remediation of dyes

Plant/Microbe synergism	Dyes	Structure and formula weight	Mechanism	Reference
*P. grandiflora and P. putida*	Direct Red 81	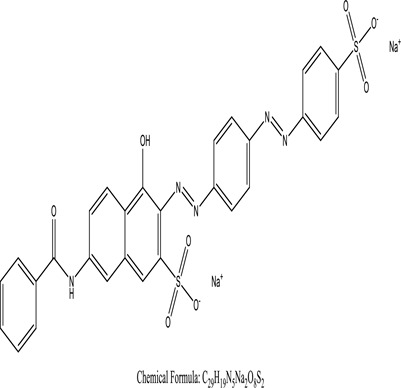	Root help in adsorption of 2,6-DCIP reductase	[Khandare, R.V., 2013]
*P. grandiflora with B. pumilus*	Reactive Blue 19	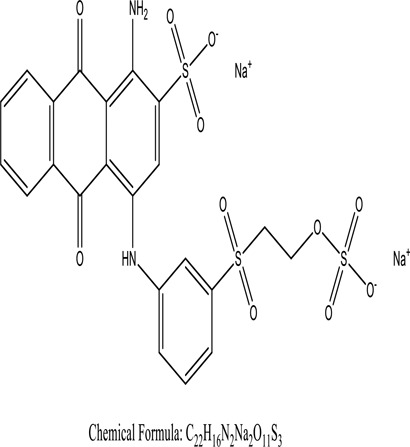	98% sorbent rate of flavin reductase activity	

### 5.4. Remediation of dye using phytoplanktons

Several macrophytes were recommended for dye degradation, and they are few to mention: *Spirodela polyrhiza, Nasturtium officinale, Paspalum scrobiculatum, Alternanthera philoxeroides* and *Typha angustifolia* [132–135]. The influence of the plant’s initial biomass (1–6 g) on the Acid Bordeaux B decolorization efficiency shows raising the plant’s initial biomass resulted in a higher ability for dye removal [136]. The increased plant biomass may result in a large concentration of internal and extracellular enzymes involved in dye breakdown, resulting in a rapid rate of dye removal [137]. Furthermore, high macrophytes abundance provides an abundance of surface areas for dye sorption [138]. For example, increasing the biomass of *Nasturtium officinale, Spirodela polyrhiza*, and *Lemna minor* (from 1 to 4 g) has been shown to increase the decolorization effectiveness of Acid Blue 92, Basic Red 46 and Direct Blue 129, and by 29%, 51%, and 58% respectively [139–141].

### 5.5. Remediation of dye using Algae

Algae are prevalent in both fresh and sea water and are now being widely explored as a biosorbent [142,143]. Microalgae plays pivotal role in the treatment of biological pollution. Its capacity to biologically purify wastewaters from a variety of sources while employing effluent as a growing medium has shown considerable promise as a sustainable and cost-effective wastewater treatment technique [144,145]. Algae have the greatest biosorption potential and electrostatic force of attraction for pollutants due to their enormous porous structure and affinity. Developing effective biodegradation strategies for microalgae is a major focus of research community [146]. Microalgae bioremediation is a relatively new technology because it is more environmentally friendly and has a smaller carbon footprint than other traditional approaches [147]. Numerous researches have shown that metabolites of toxic chemicals found in effluents, such as PO_4_^3-^, RCOO, -OH, and -NH_2,_ are digested by algae [148].

Algae decolorize the pigment in three distinct ways:

- To begin, algae collect algal biomass, CO_2_, and H_2_O via the use of chromophores;
- algae play an important role in the transition of chromophore elements to non-chromophore element;
- finally the resultant chromophores are absorbed on algae [149].

Numerous investigations have shown that the algae have more efficacies in decolorizing azo dyes by generating the azoreductase enzymatic activity [150–152]. According to certain research, algae species such as *S. rhizopus* for acid red 247, *Chlorella pyrenoidosa* for methylene blue, *N. muscorum, U. lactuca, Desmodesmus sp, Cosmarium sp, Sargassum* sp and *Pithophora sp*, potential species in degrading azo dyes into aromatic amines, which are then catabolized into simpler nontoxic forms. Several researchers have revealed that algae species use azo dyes as a source of carbon and nitrogen for growth [153]. *C. vulgaris* are applied as a natural adsorbent for removing cationic dyes. Electrostatic interaction causes the negative charged *C. vulgaris* to absorb the positively charged methylene blue [154].

*U. lactuca* is a tiny algae that is widespread across the ocean and is edible, sometimes referred to as sea lettuce. *Ulva lactuca* has been authorized as an adsorbent for remediating dye effluent [155–158] and hazardous heavy metals [159,160]. *U. lactuca*, green algae, was widely used as a biosorption for removing methylene blue dye. The capacity of *U. lactuca* to remove dye colour is time-dependent, algal biomass-dependent, dye concentration-dependent, and pH-dependent. The increased biosorption during the first contact period might be a result of the dye’s key driver onto the surface of *U. lactuca* [161]. (Table 4: List of macrophytes, structure and mechanism of dye)

**Table 4.** Macrophytes in remediation of dyes

Plant/Microbe synergism	Dyes	Structure and formula weight	Mechanism	Reference
*Sargassum glaucescens, Stoechospermum marginatum*	Naphthol Blue Black	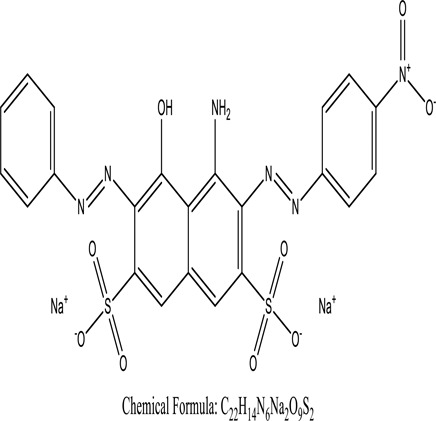	Amine groups help in binding the dye	[Daneshvar, E et al. (2012)]
*Gracilaria verrucosa*	Phenoxyalkanoic acid	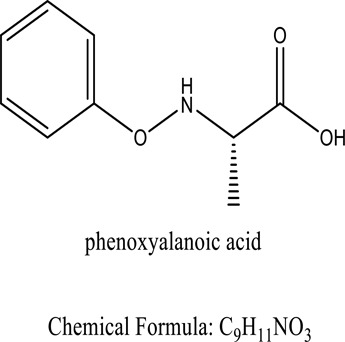	The biosorbent strength was determined to be 22.3 mg/g	[Garge MS (2012)]
*Cyanobacteria and N. limckia HA 46*	Reactive Red 198	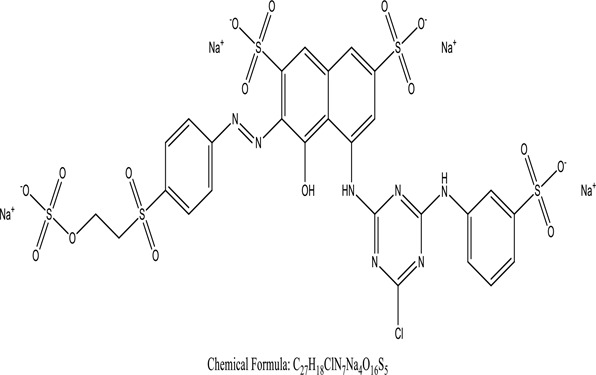	At pH 2, the biomass had a maximal sorption capacity of 94%.	
*Chlorella vulgaris*	yellow 2 G	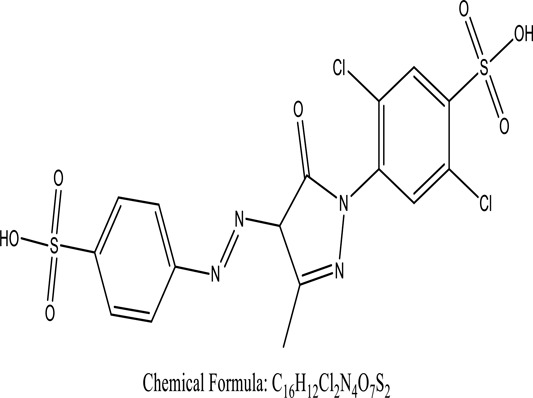	63–69% of the dark color were removed from azo dye	Aravindhan R, et al. (2007)
*Chlorella vulgaris*	Ramazol golden yellow RNL (Reactive Orange 107)	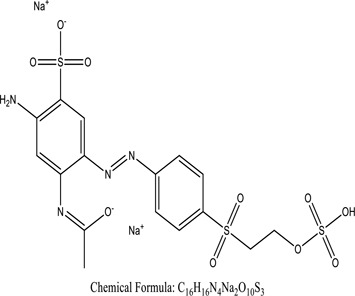	For all dyes, the obtaining maximum optimal absorption capacity is at a pH of 2.0	Aksu Z, et al. (2003)
*Anabaena hydrophila*	Reactive Blue 5	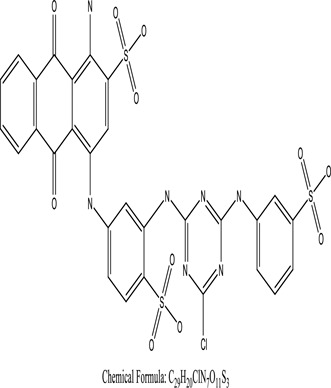	The optimum dye degradation effect was recorded at pH range of 6–9 and varied dye concentrations (5–50 mg/L)	Ogugbue, C.J., (2012)

### 5.6. Remediation of dye using Fungi

Fungi-mediated remediation has been shown to be successful in the elimination of triphenylmethane dyes [162]. Usually, remediation is accomplished by the employment of *P. chrysosporium*, multicolored *T. versicolor* [163,164]. *L. lacteus* [165], *F. solani* [166], and *P. simplicissimum* have all shown to be potential strain in dye removal [167,168]. Fungi are widely used to cultivate and provide a proteolytic enzyme that is effective for color degradation [169,170]. They produce enzymes that naturally degrade hazardous dye compounds into less or harmless simplified variants [171]. Coriolopsis sp. (1c3) has been reported to decolorize MG, CB, CV and MV with 52, 91, 94, 52, 97% decolorization respectively [172]. It is successively studied that *Aspergillus niger, Aspergillus oryzae*, and *Rhizopus arrhizus* is capable of removing acid orange 7 dye with a stability of 9.97, 9.76, and 11.43% in a neutralized aqueous media. This is because the amino groups on the chitosan molecules on the attenuated fungal cell wall were positively charged, resulting in positively charged – NH_3_^+^ groups that are electrostatically attracted to the acid orange 7 dye. The adsorption of acid orange 7 dyes by dead fungal cells was at low pH. Instead of using free mycelium, the administration of Coriolopsis (1c3) sp. in biofilm form was more effective, resulting in a much higher level of Crystal violet and Cotton blue removal. The decolorization of CB and CV was 79.6 and 85.1% respectively, with the application of biofilm [173]. *Aspergillus carb*onarius, a dead biomass, is an efficient quencher of hexavalent chromium from e-waste polluted water [174]. (Table 5: List of Fungi, structure and mechanism of dye).

**Table 5.** Fungi used in remediation of dyes

Plant/Microbe synergism	Dyes	Structure and formula weight	Mechanism		Reference
*T. polyzona*	Bisphenol Bromophenol Blue	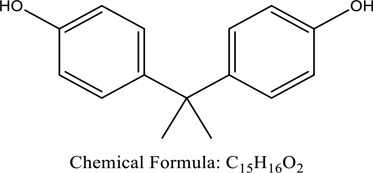 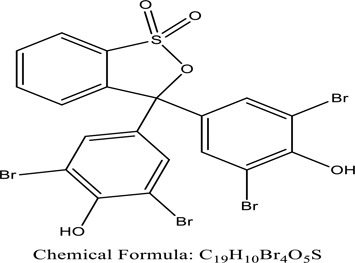	Root help in adsorption of 2,6-DCIP Reductase Rapidly oxidized bisphenol		Chairin, T (2013)
*A. bisporus* *T. orientalis*	Reactive blue 49	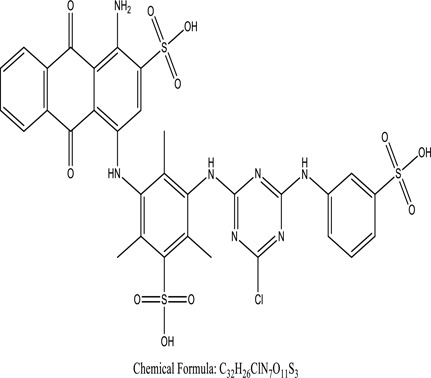	Combined adsorption capacity was 72.86mgg− 1	[Akar, S.T et al. (2009a)]	
*Rhizopus arrhizus*	Direct Yellow 86	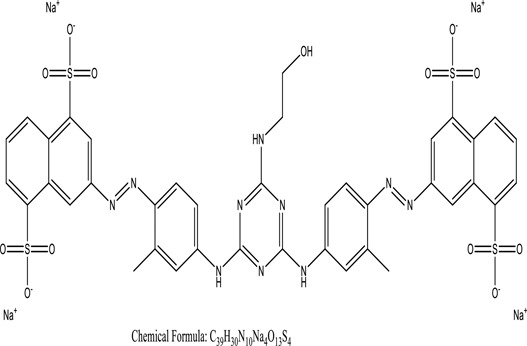	Metal-complex dye biosorbed by 85.4-mg dye g − 1	Aksu, Z., et al. (2010)	
*Aspergillus fumigatus*	Methylene blue	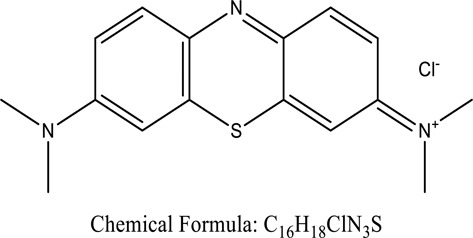		Kalyani, P., et al. (2017)	
*Aspergillus fumigatusXC6*	Reactive Yellow 3	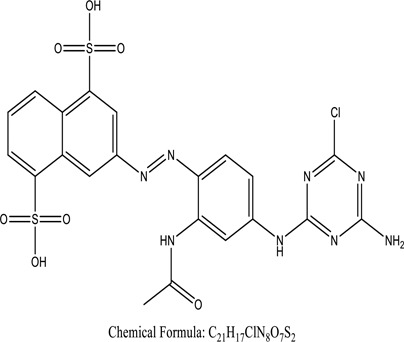	While nourished with 1% sucrose, the strain destrain the discharge at initial pH	Xian-Chun Jin. et al. (2007)	
*Phanerochaete chrysosporium*	4-Nitrotoluene	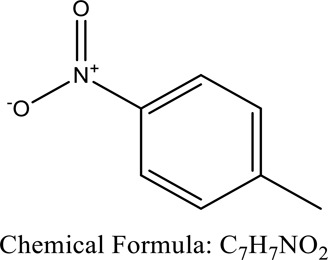	Capability of partly or effectively degrading recalcitrant organic contaminants	Barr D. P et al. (1994)	
*Trametes versicolor*	Indigo carmine	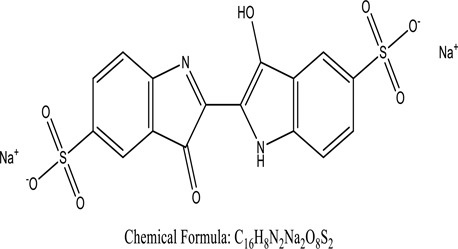	Laccase was the enzyme responsible for dye degradation	Wong, Y. (1999)	

### 5.7. Remediation of dye using Yeast

Many studies have utilized yeasts to breakdown dye from effluents. *Debaryomyces polymorphus* has been used to breakdown the dye Reactive Black 5 [175], while several yeast species isolated from tropical rainforests, including as Trichosporon, Cyberlindera, Barnettozyma, and Candida, have also been used to breakdown colors [176]. Baker<apos;>s yeast has also been used recently to degrade Astrazone basic dye [177] *Galactomyces geotrichum* MTCC 1360 was shown to have an 88 percent removal efficiency in mixes of structurally distinct dyes (Remazol Red, Golden Yellow HER, Rubine GFL, Scarlet RR, Methyl Red, Brown 3 REL, and Brilliant Blue) [178]. *Staphylococcus epidermidis* was used to breakdown Crystal Violet, Phenol Red, Malachite Green, Methyl Green, and Fuchsin into non-toxic compounds [179]. Moreover, a comprehensive investigation on the isolation of yeasts and their capacity to breakdown diverse colors was reported [180]. The yeast *Saccharomyces cerevisiae* is often used as a biomaterial in textile wastewater remediation [181]. The elimination of methylene blue (MB), a reactive dye, was investigated using *Saccharomyces cerevisiae*, on the other hand, significantly reduces the color absorbance and COD value of azo dyes, ramazole blue (Vinyl sulfone), by 100% and 61.82 percent, accordingly [182]. The use of yeast as a mediator for adsorbing congo red and methylene blue demonstrated that electrons were transported to anode from the substrate through the dyes, resulting in the generation of electrostatic force. MOP<apos;>s high ability for removing CR paves the way to the development of a high-performance biosorbent for the removal of anionic dyes from aqueous environments [183]. For the treatment of industrial waste, the adsorbent containing *Brevibacillus parabrevis* bacteria holds great potential [184]. The energy generated by the fuel cell was then used to remove traces of potential lead from a dilution water solution [185]. *Candida tropicalis* had the capacity to adsorb basic violet 3, and this is due to the smallest particle size (150–300 µm) and larger surface area [186].

### 5.8. Microbial remediation

Microbial degradation has been extensively explored and evaluated, mostly with the purpose of enhancing dye degradation [187]. Microorganisms play a critical role in the full breakdown of dyes. Microbial degradation of dyes has been proved to be very effective for resource recovery and sustainability [188]. Various microbes have already been identified as bioremediator in various industries [189–193]. Microbes based researches have been published using a variety of microorganisms in liquid and consortiums culture [194–197]. Adsorption of synthetic dyes using laccase enzymes from *P. rubidus, B. juncea, T. versicolor* and *T. hirsuta* [198–201] and lignin peroxidase enzymes from *B. laterosporus* MTCC 2298 show 90% degradation potential. There is a surge for bio remediating techniques in waste disposal. So, there is a need to develop new and innovative procedures for the effective and environmentally friendly disposal of diverse kinds of pollutants at a low operating cost.

### 5.9. Genetically modified organism in bioremediation

The introduction of a desired gene of interest into a microbe for a specific reason that is not normally found in the target host results in a genetically modified organism. Although the environment has a self-cleaning mechanism in response to climate and ecological stress, there is evidence that it would be inadequate and sluggish to remove contaminants [202,203]. Numerous chemical, physical and biological methods for the elimination of toxic chemicals including dyes have been explained. These methods may be applied alone or in combination [204–206]. Nowadays, toxic chemicals from dyes can be easily removed by genetically modified microbes, which will have high resistance for pH, light and temperature, but it is time consuming and labour intensive technique [207]. Each genetically modified microbe is unique in its capacity to degrade, detoxify, and decolorize dyes. GMOs are the most often utilized organisms in bioremediation with zero toxic discharge in water bodies [208].

Genetic modification has revolutionized the concept of bioremediation [209]. Under certain climatic circumstances, it is possible to enhance dye removal by employing genetically engineered microorganisms. GMOs may be created by genetic modifications across species or via genetic manipulation [210–213]. To create GMOs, functional genes from a variety of bacteria were isolated from *R. eutropha, B. idriensis, P. putida, M. marinum, E. coli* and *S. desiccabilis*. The organism modified showed the elimination of toxic chemicals, including synthetic dyes [214]. Many innovative techniques were available to determine microbial genome expression, including polymerase chain reaction (PCR), single-stranded conformation polymorphism, 16S rDNA sequencing, randomly amplified polymorphic DNA and other emerging sequencing technologies [215–217]. Genetically modified *E. coli* SS125 were used for the breakdown of Remazol red dye by cloning the azoreductase gene from *B. latrosporus* RRK1 into *E. coli* DH5a and pAZR-SS125 [218]. Engineered *E. coli* JM109 (pGEX-AZR) strain in the laboratory that decolorizes direct blue 7[219]. Remazol red may be degraded in the presence of 0.8 mg/L of O_2_ using the azoreductase gene from *B. latrosporus* RRK1 and inserted into *E.coli* [220]. To break down and denature triphenylmethane dyes, a novel consortium of four strains namely *A. hydrophila, A. radiobacter, Bacillus sp* and S. *paucimobilis* [221], were used. CV and MG were triphenylmethane color are employed in dyestuff industry sectors and in the making of printing paper were successfully degraded using the above mentioned 4 novel consortiums [222–224]. Certain TPM dyes are xenobiotic chemicals, which are commonly regarded as a major source of environmental contamination [225,226]. The mutagenicity of CV and MG were degraded using *Salmonella typhimurium* TA98 and TA100. The bacterial consortium has been proven as one of the vital techniques to be used in dye industries [227] (Table 6: Bacteria/ Bacteria Consortium used in the remediation of dyes).

**Table 6.** Bacteria/ Bacteria Consortium used in the remediation of dyes

Plant/Microbe synergism	Dyes	Structure and formula weight	Mechanism	Reference
*A. caviae*, *P.mirabilis* *R. globerulus*	Acid Orange 7	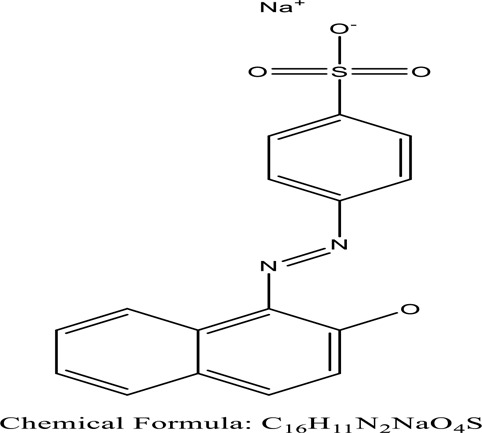	Even at 200 mg/l, 90 percent decolorization may be accomplished after 16 hours	[Joshi T, et al. (2008)]
*Bacillus gordonae*, *Bacillus benzeovorans*, *Pseudomonas putida*	Acid Blue 277 (Tectilon Blue)	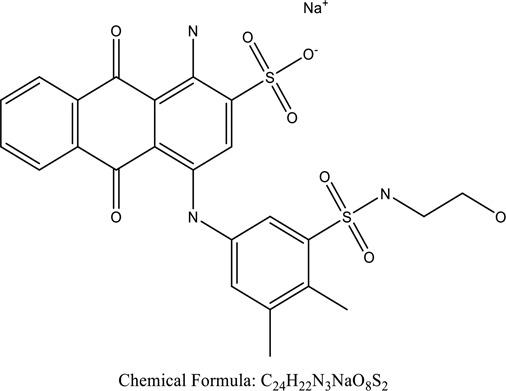	Dye degradation is accurately simulated during a 24-hour at a response rate of 200–1000 mg/l	[Walker et al. (2000)]
*B. subtilis* *E. coli Azotobacter* *Providencia sp. SRS82*	Acid Black 210	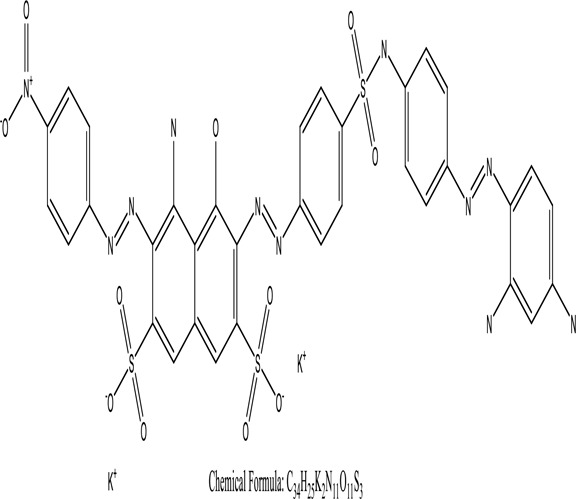	Under optimized conditions, 100 mg/L dye degrades in 90 minutes	Agrawal et al. (2014)
*P. polymyxa* *Bacillus polymyxa* *Micrococcus luteus*	Reactive Violet 5 R	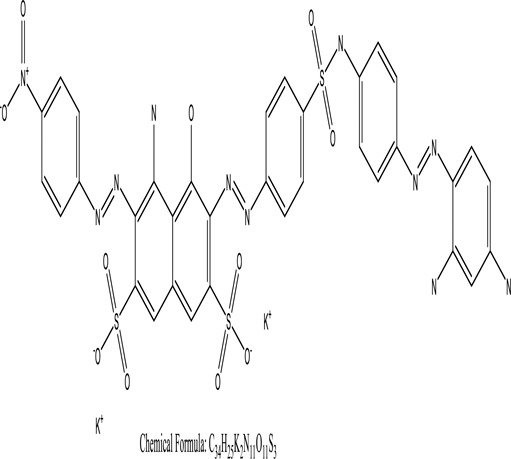	Within 37 hours, it demonstrated a 94 percent decolorization ability in alkaline pH	Moosvi, S et al. (2005)
*Alcaligenes faecalis, Sphingomonassp. Bacillus subtilis, Bacillus thuringiensis*	Direct Blue-15	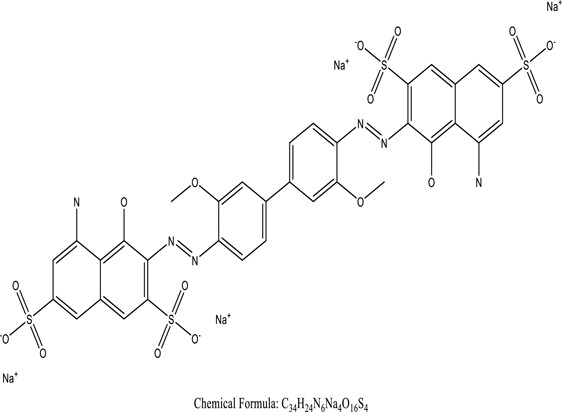	Most capable of decolorizing at alkaline pH at 30°C	Kumar K (2009)
*Proteus vulgaris* *Micrococcus* *glutamicus*	Scarlet R	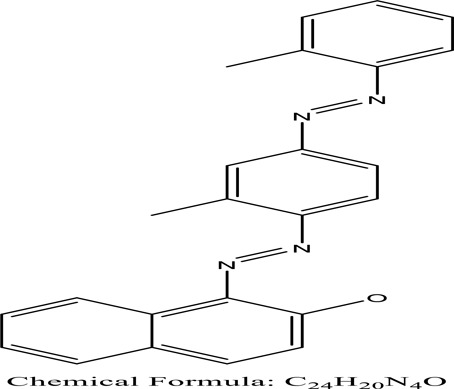	After 3 hours, a decrease of over 90% in TOC and COD	Saratale RG et al. (2009)
*Bacteroidetes Firmicutes*		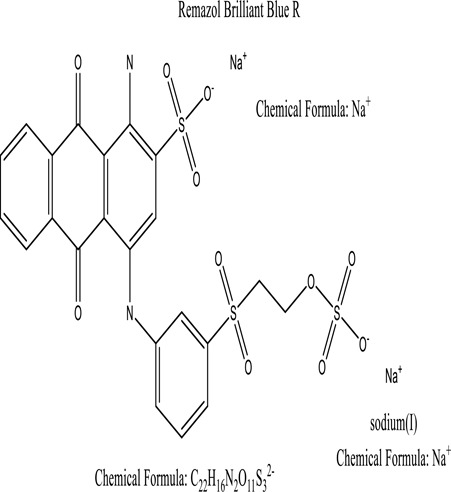	The CODCr elimination rate, the BOD5/CODCr value, and the synthesis of volatile fatty acids (VFAs) all were almost 95% successful	Liu, N., et al. (2016)
*Bacillus thuringiensis SRDD*	Acid Red 119	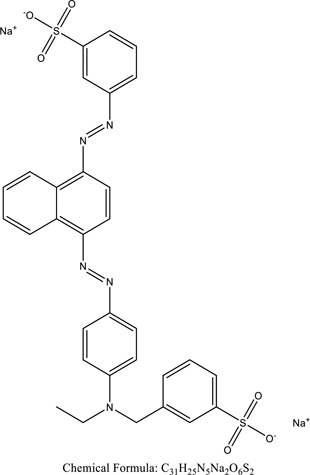	Exhibited decolorisation up to 1000 ppm of AR-119 dye after 7 days of observation	Dave SR, Dave RH (2009)
*P.aeruginosa NGKCTS*	Reactive R111	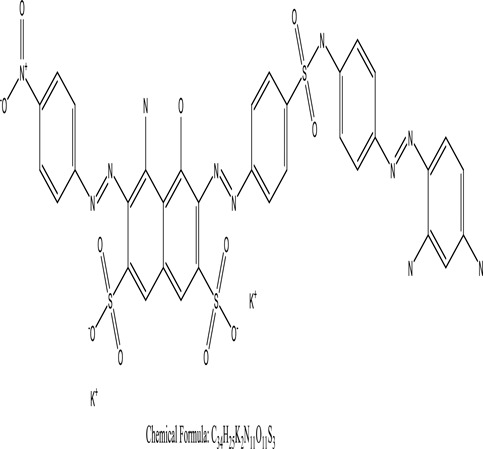	Within 5.5 hours, 91 percent of 300 ppm dye was decolorized across a wide pH range	Sheth, N.T., et al. (2009)
*Sphingomonas herbicidovorans FL*	Bromaminic Acid	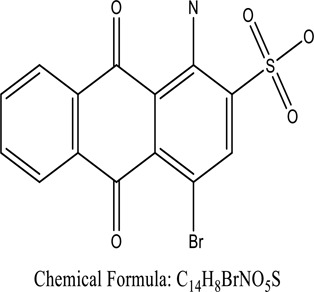	98% within 24 h even for the initial concentration greater than 1000 mg l-1	Fan L et al. (2008)
*Pseudomonas sp. strain DY1*	Acid Black 172	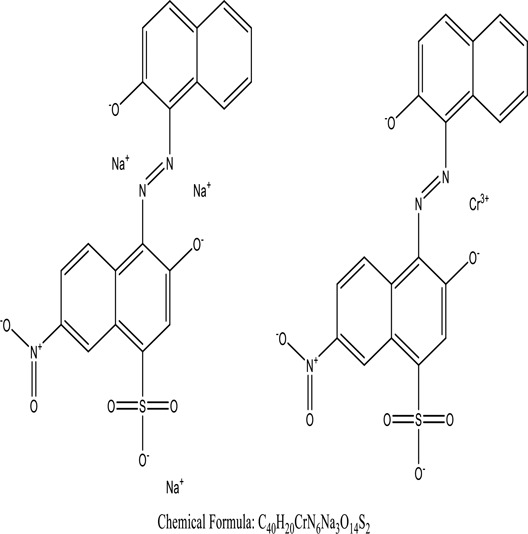	Adsorption of dyes reached a maximum of 2.98 mmol/g biomass	Du LN, et al. (2012)
*Pseudomonas aeruginosa 23N1*	orange 16 Reactive red 21	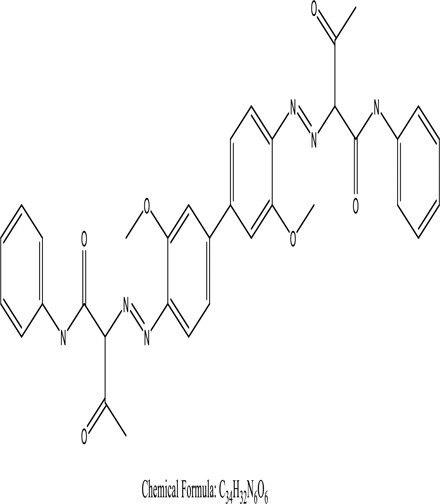 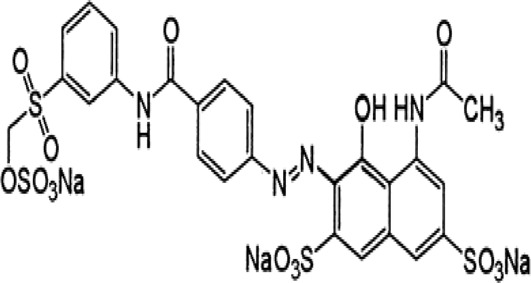	Exhibit satisfactory ADMI reduction	Mishra, S., et al. (2020)
*Citrobacteria CK3*	Reactive red 180	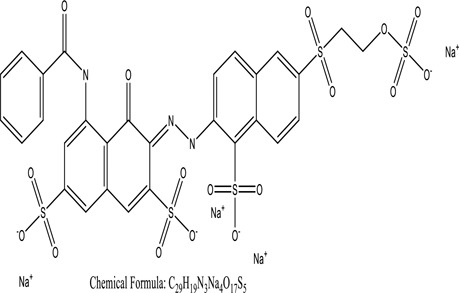	Decoloration (96%)	Wang (2009)
*Klebsiella strain Bz4*	Brilliant Green dye	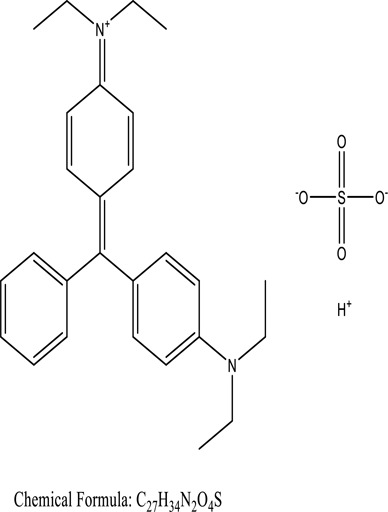	Following 24 hours of treatment, 81.14 percent of the dye has been removed, and after 96 hours, 100 percent of the dyes were removed	Zabłocka-Godlewska, et al. (2015)
*Salinivibrio kushneri HTSP*	Coomassie brilliant blue (CBB)	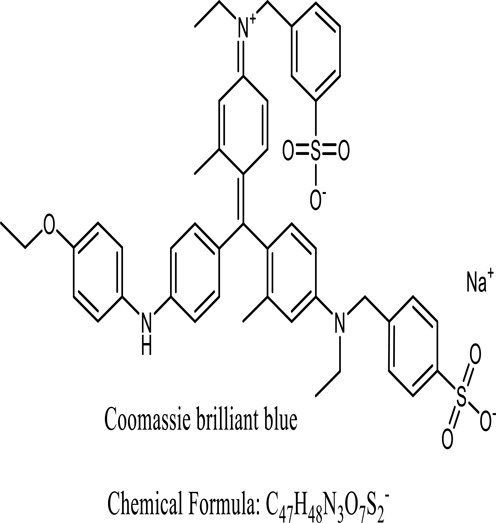	After 48 hours, over 80% of dye removal was seen	John J, et al. (2020)
*Halomonas elongate* *Shewanella oneidensis MR-1*	Methyl red	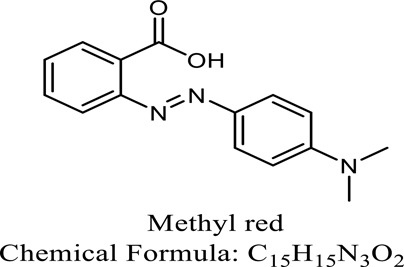	Methyl red has a specific outcome of 0.27 mol min1 mg1	Eslami (2016); Cao (2017)

### 5.10. Bioflocculants in dye removal

Bacteria capable of creating bioflocculants are widely separated from wastewater treatment plants. The bioflocculants derived from indigenous microorganisms were extremely successful in decolorizing the various colours. Bioflocculants are used in many industries including treating wastewater, household, brewery, and pharmaceutical wastewater treatment, textile manufacturing, sewage treatment systems, and cosmetics processing [228,229]. Bioflocculants generated by strains xn11 + xn7 were successful in eliminating the basic fuchsin (100 mg L1) but comparatively less efficient at decolorizing reactive black (50 mg L1), with dye removal efficiencies of 93 and 95%, respectively [230]. Due to their low cost and ease of application, biological approaches have become the subject of interest on dye degradation and decolorization [231]. Bioflocculants generated by *B. subtilis* (E1), *E. acetylicum* (D1), *K. terrigena* (R2), *S. aureus* (A22), *P. pseudoalcaligenes* (A17), and *P. plecoglossicida* (A14) were capable of decolorizing textile industrial effluent with maximum adsorption. Fungus *F. carnea* was used as a bioflocculant, that enhanced the reduction and removal of three cationic dyes namely Orlamar Red BG, Orlamar Blue G, and Orlamar Red GTL [232]. Bioflocculant *Rhizopus arrhizus* was used to degrade Remazol Black B reactive dye at optimal adsorption temperature 35°C.Due to decreased surface activity, there was a decrease in adsorption as the temperature increased [233,234].

Cations promote flocculation by neutralizing and stabilizing functional groups’ residue negative charge and by establishing links interconnecting particle. Divalent and trivalent cations promote the initial sorption of biopolymers on suspended solids by lowering the negative charge on both the polymer and the particle [235]. Mn2^+^, Mg2^+^, and Ca2^+^ have been found to form complexes with bioflocculants, so increasing flocculation and decolorization [236]. However [237,238], demonstrated that the presence of any cation, including Ca2+, did not improve the flocculating activity of Citrobacter sp. TKF04 and *G. impudicum* KG03. Due to the high salt content in dyeing operations, the salt concentration in dye-containing effluent is a critical factor affecting biosorption ability [239]. Flocculants may remove dyes (anionic azo-dyes) by neutralization of charges as well as by bridging effects, with the former being the primary mechanism [240,241]. The dye functional elements seem to favour new interactions, which results in the development of insoluble dye which may be precipitated. Furthermore, the efficacy of decolorization by microbial bioflocculants is highly dependent on the kind of dye, pH, exposure to light and flocculation concentrations.

## 6. Recommendation and future perspectives

Although bioremediation had already established as an effective treatment option for water purification, various obstacles prevent its widespread commercial applicability. The current practices must be resolved in order to maximize the significance of bioremediation technologies in industrial wastewater treatment [242].

- Future research on dye degradation should focus on reducing the challenges posed by constraints on plants and microorganisms.
- Recent and early successful research must be re-examined to optimize their effectiveness.
- A biodegradation method that is effective should take into consideration degradation pathways, environmental conditions, interfacial properties, and degradation processes that impact pollutant removal.
- It is vital to ensure that the degraded products do not pose a threat to aquatic life or vegetation.
- The notable intent of the research was to create marine psychrophilic bacteria with novel and unique biodegradation capabilities for the biosorption of chemically polluted cold environmen.

**Figure 5.**
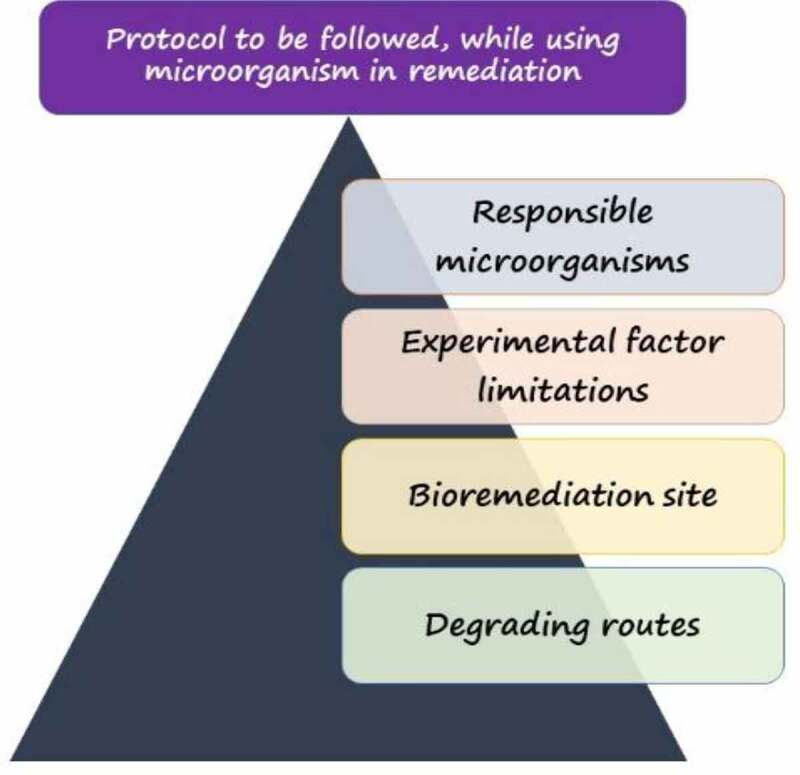
Protocol to be followed to achieve remarkable dye biodegradation.

The investigation of the processes and hypotheses behind bacterial degradation of dye wastewater would benefit the exploration of bacterial degradation kinetics (Figure 5).

## 7. Pros and cons of plant microbe based dye remediation

**Pros**:

- Despite certain limitations, phytoremediation and microbial remediation is mostly beneficial and may be incrementally improved using contemporary biotechnology approaches including the development of more degrading and resistant engineered organisms.

**Cons**:

- Effective in removing contaminants at low volumes and concentrations. Extremely excellent for removing certain colors
- Resistant against a broad range of colored chemicals with a complicated structure.

## 8. Conclusions

Discharge of textile industry effluents to natural water bodies (such as natural ponds, rivers, creeks, streams, and river systems) may be classified according to the presence of non-degradable colors and hazardous compounds. This chapter discussed the environmental impacts of dye contamination caused by some dye industries, as well as the many techniques employed by plants and bacteria to efficiently remediate polluted reservoirs and ecosystems. It is found that the use of bioremediation will be cheaper, and efficient for removing dyes from polluted water bodies. It is also cost-effective than the traditional than the physico–chemical approaches, which take higher energy. Microorganisms, yeast, fungi and plants are endowed with biological mechanisms that enable them to survive under synthetic dye stress and degrade the components to a less toxic or non-toxic state. These bacteria use a variety of activities, including precipitation, adsorption, enzyme-mediated ion transformation, sorption, and bioconversion strategies, in which the most successful techniques are phyto-extraction and phyto-volatilization. Furthermore, the changes in the environment must be favorable for bioremediation to be effective. The application of biosorbents plants and microbes to polluted water bodies is dependent on the level of dye present and the kind of aqueous solution. Ecological variables are important for bioremediation effectiveness, since the microorganisms that were used will be killed in presence of unfavorable environmental conditions. Particularly fast-growing flora with a larger efficiency for phytoextraction should be identified for treating wastewater. Additionally, a study of the impact of dye stress on beneficial endophytic bacteria should be performed, and efficient methods for increasing the bioremediation process should be recommended. While transgenic microorganisms and plants have the potential to efficiently remediate dye and organic pollutant-contaminated environments, their usage should be subject to severe biosafety standards to guarantee that there are no health or environmental risks. Improved effective methods of using transgenic plants and bacteria should be identified that would enable successful restoration of contaminated habitats without the need for horizontal transfer of recombinant plasmids to indigenous species, which is presently a significant barrier.

Genetic engineering is an emerging field of study that will support the development of synergetic microbes capable of degrading and removing colours from industrial effluents through the metabolic features of these consortia of organisms. This technique should be encouraged to enable more effective pollution treatment. So, plant and yeast microbial-based wastewater treatment techniques have now been achieved utilizing microbial consortia or a single dye-degrading microbial strain. However, metagenomic and enzymatic techniques must also be employed to investigate the functional makeup of bacterial diversity inside the polluted locations. The metal resistance genes that may be utilized to enhance particular heavy metal degrading strains of microorganisms. These concerns the adoption of alternative green technologies for the remediation of harmful synthetic chemicals found in wastewater.

## Highlights

- Dyes play a pivotal role in our everyday life.
- Categorization, structure, and degradation of dyes in textile wastewater effluent.
- Critiques of several physicochemical factors on the dye removal effectiveness
- Emphasizes on plant metabolic and extractive ability to deal with colorant
- Readers will get insight into the future prospects and pitfalls of remediation.
